# Synthesis and crystal structure of *N*-benzyl-5,6-ep­oxy-7-oxabi­cyclo­[2.2.1]heptane-2,3-dicarboximide

**DOI:** 10.1107/S2056989026009205

**Published:** 2026-09-08

**Authors:** Joseph F. DeJesus, Mary Helene Marmande, Bailey N. Baxter, Masakazu Nambo, Cathleen M. Crudden, David C. Forbes

**Affiliations:** a Institute of Transformative Bio-Molecules (WPI-ITbM), Nagoya University, Nagoya 464-8601, Japan; b University of South Alabama, Department of Chemistry 6040 USA Drive South Mobile Alabama 36688 USA; c Queen’s University, Kingston, Ontario K7L 3N6, Canada; University of Massachusetts Dartmouth, USA

**Keywords:** polycyclic, epoxide, synthesis, stereochemistry, norcanthardin, crystal structure

## Abstract

The title compound, C_15_H_13_NO_4_, is an analog of a well-known cytotoxic mol­ecule, canthardin. The compound possesses a 3/5/5/5 contiguous stereo-defined ring network, which features cyclic furan, oxirane and cyclic carboxamide moieties. The flexibility of the *N*-benzyl unit allows for supra­molecular chiral (but racemic) packing in the extended crystal structure. The inter­molecular inter­actions in the crystal are dominated by H⋯H and O⋯H van der Waals inter­actions, as shown by Hirshfeld analysis.

## Abstract 2

The crystal structure of the title compound demonstrates a tetra­cyclic organic compound with defined stereochemistry. In the solid state, the compound organizes in a racemic chiral fashion with alternating *N*-benzyl units.

## 1. Chemical context

**Figure 1:**
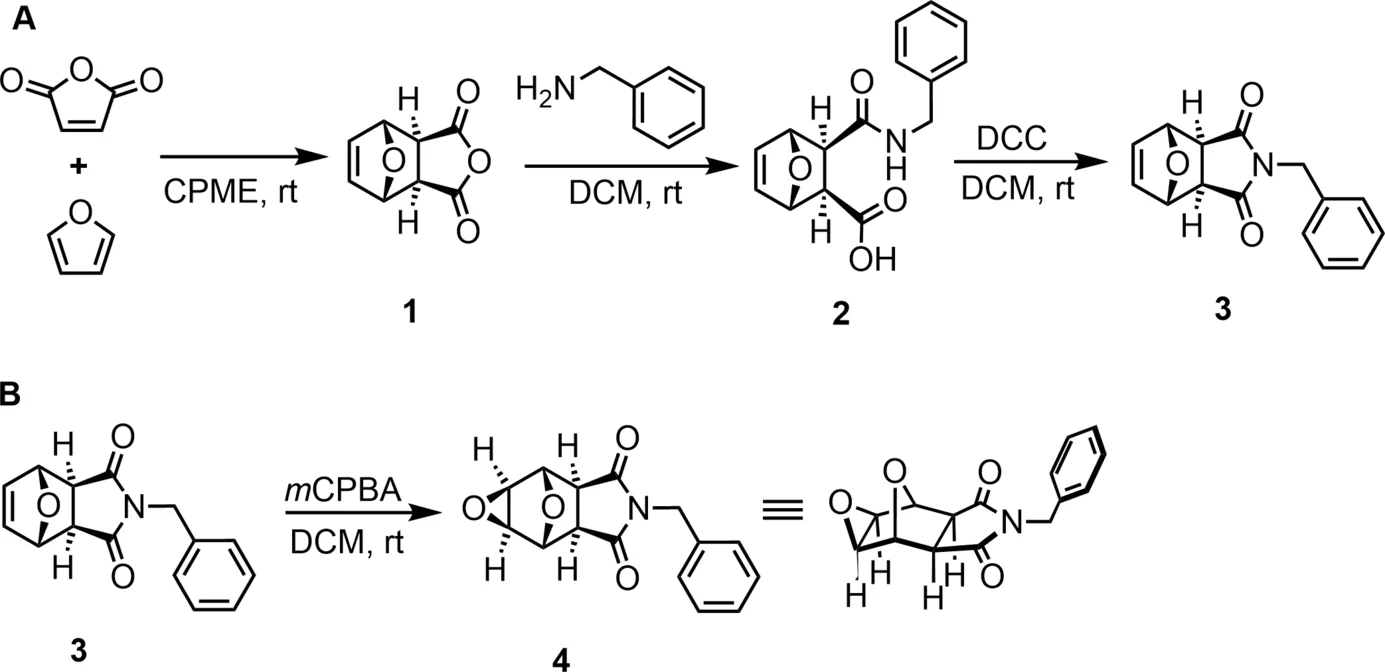
(A) Synthesis of alkene synthon of 7-oxabi­cyclo­[2.2.1]hept-5-ene core (**1**) and transformation of anhydride moiety into more stable 2,3-dicarboximide group (**3**). (B) Transformation of **3** to **4** using *m*CPBA oxidation, and its three-dimensional arrangement with conserved stereochemical control.

Compounds based on the 7-oxabi­cyclo­[2.2.1]heptane motif have attracted considerable attention in medicinal chemistry because their conformationally constrained structures can effectively mimic biologically relevant architectures (Scott *et al.*, 2026 ▸). This framework is closely related to those present in norcantharidin, a system whose methyl­ated cousin cantharidin has been investigated for anti­cancer, enzyme-inhibitory, and agrochemical applications (Lawley *et al.*, 2026 ▸). Norcanthardin derivatives also possess the advantage of simplicity and scalability of synthesis, for example, by a very straightforward Diels-Alder cyclization (Fig. 1 ▸, formation of **1**). In our hands, compound **1** can be isolated in 76% yield in gram qu­anti­ties by this route, with exclusive *exo* selectivity (Hill *et al.*, 2022 ▸). In the 7-oxabi­cyclo­[2.2.1]hept-5-ene core, there is a convenient point of unsaturation to allow for further derivatization (Alves *et al.*, 2023 ▸). Using **1** as a common synthon, we were inter­ested in developing new analogs of norcanthardin to study their biological effects.

This report details the formation and crystallization of the new norcanthardin analog *N*-benzyl-5,6-ep­oxy-7-oxabi­cyclo­[2.2.1]heptane-2,3-dicarboximide (**4**). Fig. 1 ▸A details the route, which includes nucleophilic ring opening of the anhydride moiety of **1** with benzyl amine to form amidic acid **2**, in 91% yield, and subsequent closing to form the phthalimide **3** with carbodi­imide chemistry (79% yield). Compound **3** is a norcanthardin analog with a 5/5/5 tricyclic rigid core and an alkene synthetic handle. Formation of an oxirane moiety through the use of *m*CPBA oxidation chemistry (Fig. 1 ▸B) resulted in formation of the highly rigid 3/5/5/5 tetra­cyclic norcanthardin derivative **4** in 97% yield.
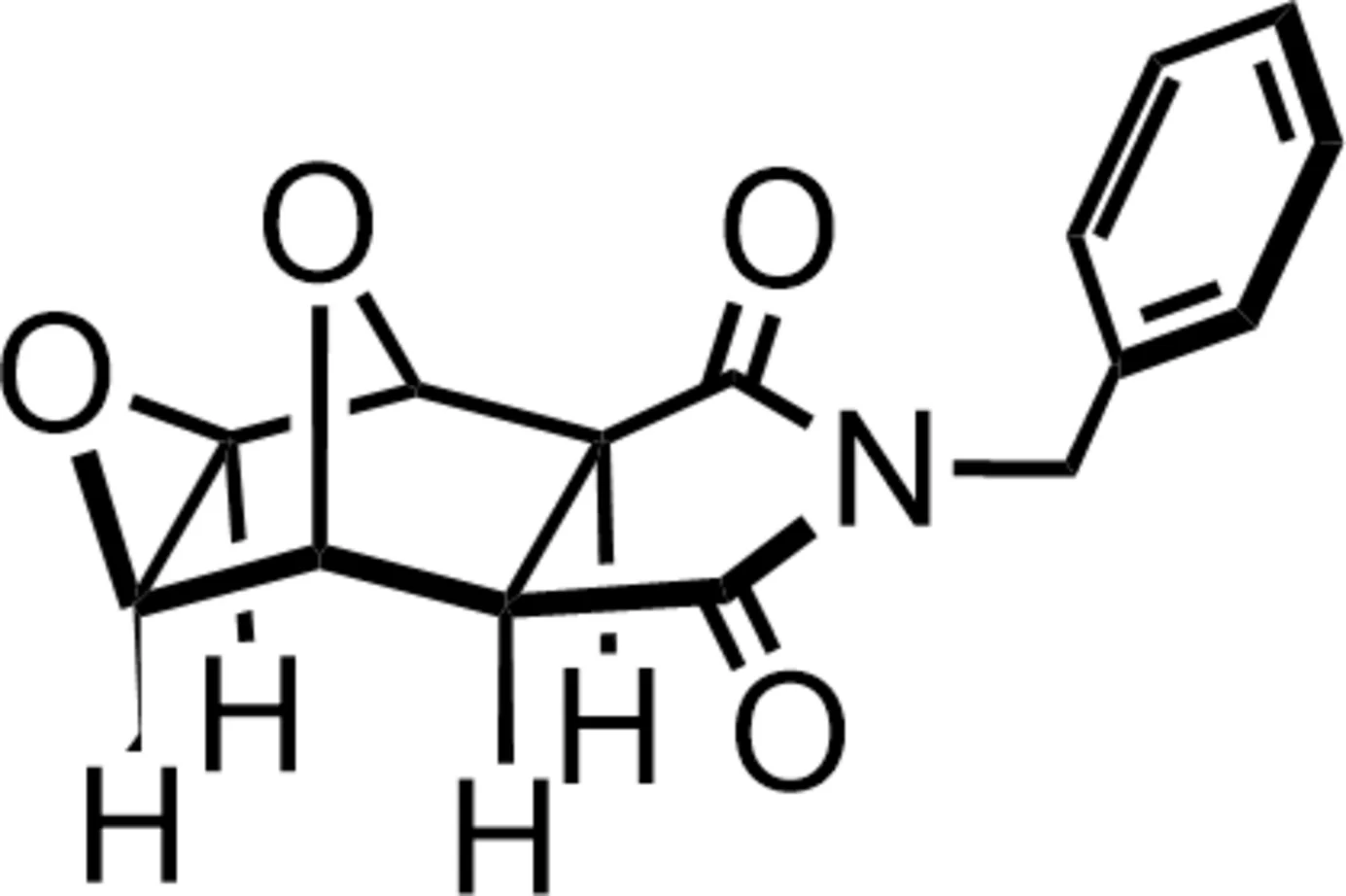


The symmetry of the 3/5/5/5 tetra­cyclic core and the use of an oxirane functionality in **4** represents both a valuable synthetic inter­mediate and a versatile platform for further development of biologically active mol­ecules. Furthermore, the rigid scaffold also imposes a well-defined three-dimensional arrangement of functional groups, making this synthon an attractive system for studies of structure–activity relationships and mol­ecular recognition. As such, we sought to characterize the solid-state properties of **4** through X-ray crystallography as an initial attempt to probe structural properties of this new analog.

## 2. Structural commentary

**Figure 2:**
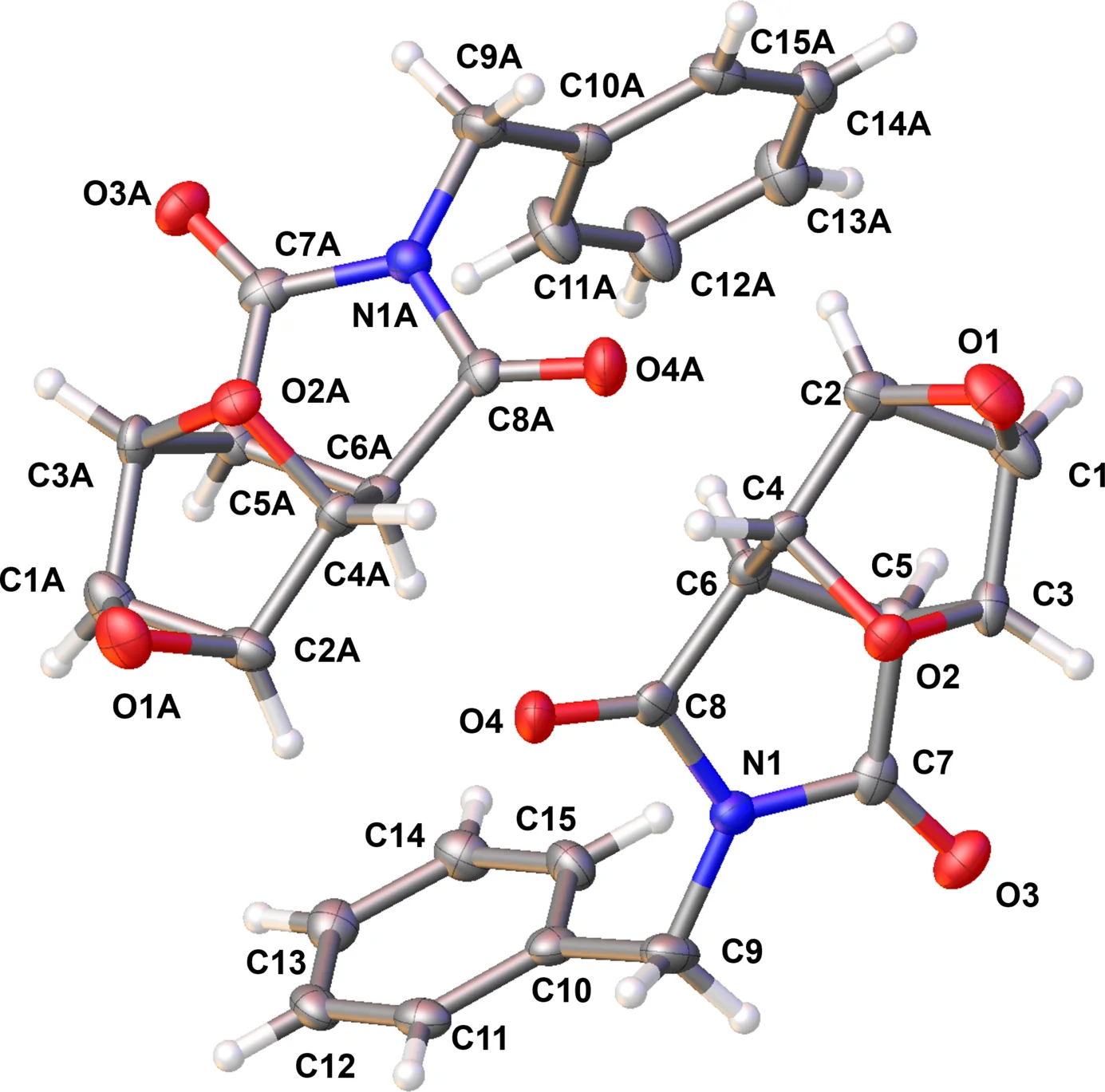
Observed asymmetric unit of **4**, with co-crystallized mol­ecule **4A**. Atom labels for each fragment are shown. Displacement ellipsoids of gray, blue and red correspond to carbon, nitro­gen and oxygen, respectively. White spheres correspond to hydrogen atoms.

Compound **4** was crystallized by vapor diffusion of hexa­nes into a 1.5 mL tube of **4** dissolved in ethyl acetate. Large colorless block-like crystals grew on the sides of the wall of the glass tube. After mounting one of these crystals, the mol­ecular structure of **4** was obtained as shown in Fig. 2 ▸. The asymmetric unit of **4** crystallizes in the ortho­rhom­bic *Pca2_1_* space group, and has two crystallographically distinct mol­ecules, **4** and **4A**, with no co-crystallized solvent mol­ecules observed. In the course of refining the structure, we found that the absolute configuration of the structure could not be determined reliably. After conducting a Bijovet pairs analysis, we found that the crystal had a 70.1% chance to be an inversion twin. As a result, the crystal was refined as an inversion twin to remedy this issue for further analysis. The rationale for formation of an inversion twin became apparent upon observing the extended crystal structure (*vide infra*, Fig. 4).

**Figure 3:**
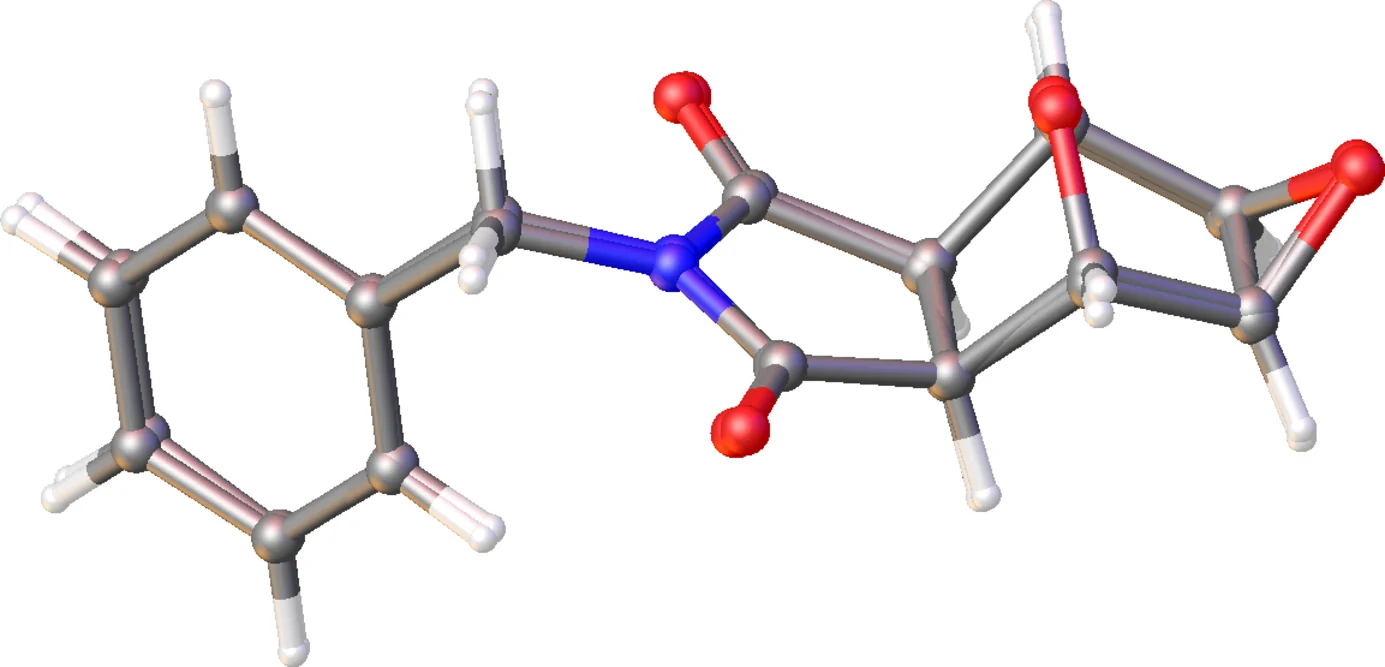
Overlayed mol­ecules in asymmetric unit, **4** and **4A**. Both mol­ecules in the asymmetric unit are nearly identical. Probability ellipsoids shown at 50%.

Overlaying both mol­ecules in the asymmetric unit (Fig. 3 ▸), demonstrates that each fragment is nearly identical, with an alignment RMSD of 0.129 Å. Of note, both fragments have the same contiguous absolute confirguration in the chiral centers (C1/C1*A* = *S*, C2/C2*A* = *R*, C3/C3*A* = *S*, C4/C4*A* = *R*, C5/C5*A* = *S*, C6/C6*A* = *R*), demonstrating that there was no erosion of the starting chirality in the formation of the oxirane unit, or during any of the prior chemical transformations. This results in all four rings of the tetra­cyclic core ‘puckering up’, and allowing the oxygen heteroatoms to orient themselves in one direction, which is critical in the cytotoxicity of this family of compounds (Chattopadhyay *et al.*, 2016 ▸). Additionally, the ^1^H NMR spectrum implies that there is only one single stereoisomer present in the bulk of the isolated compound (see Section 5, preparation of **4**).

## 3. Supra­molecular features

**Figure 4:**
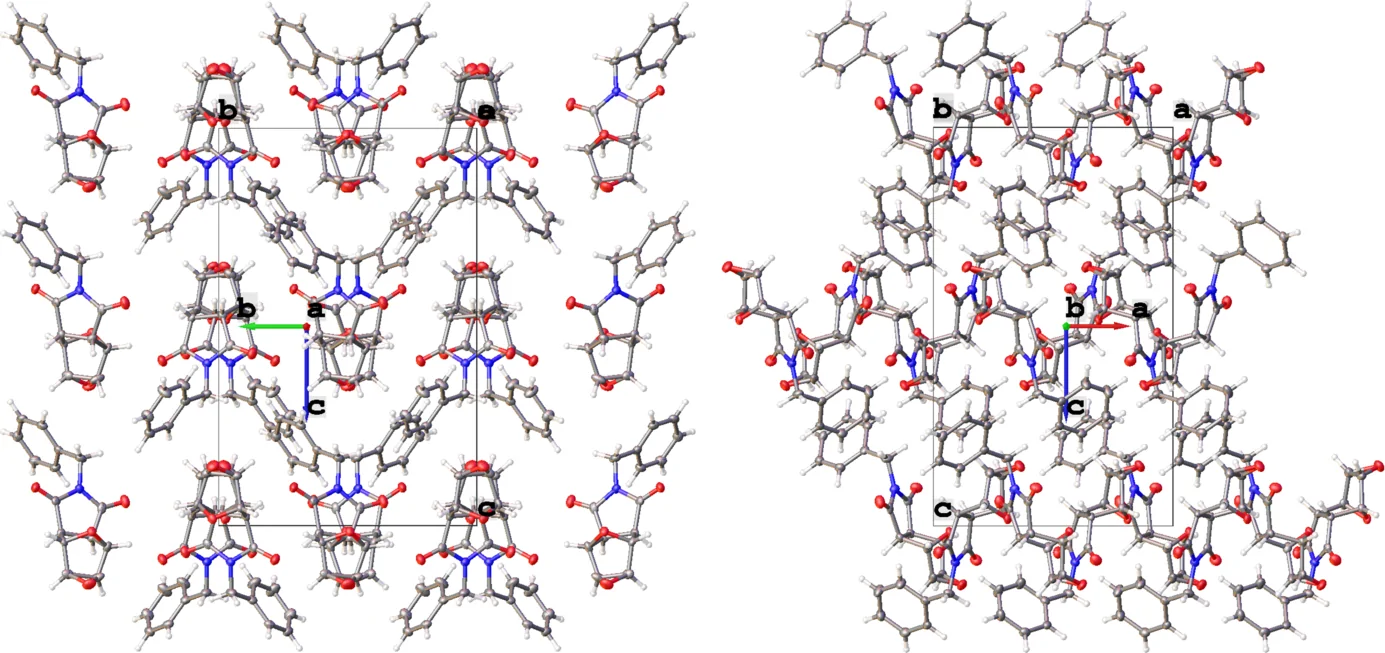
Supra­molecular packing of **4**, in a completed unit cell. Left: view of the supra­molecular packing looking down the *a* axis. Right: view of the supra­molecular packing looking down the *b* axis. Displacement ellipsoids are shown at 50% probability.

Fig. 4 ▸ shows the supra­molecular packing of **4** and **4A** down the *a* and *b* axes. Implied by the *Pca2_1_* space group, the supra­molecular structure shows the two glide-plane operations, where the mol­ecules propagate in a twisting fashion across the crystal. On the edges of the diagram in Fig. 4 ▸ left, the *N*-benzyl units are oriented in opposite directions. This alternating orientation of the *N*-benzyl units is shown in both views in Fig. 4 ▸ as an apparent packing in the crystal. These alternating *N*-benzyl units explain the two crystallographically distinct mol­ecules in the asymmetric unit, despite the individual mol­ecules being chemically identical when overlain (Fig. 3 ▸), since they possess different orientations in the expanded crystal. Because this propagation has a chiral operation, the crystal of **4** crystallizes as a racemic twin due to the presence of the opposite orientation co-crystallizing with this mol­ecule.

**Figure 5:**
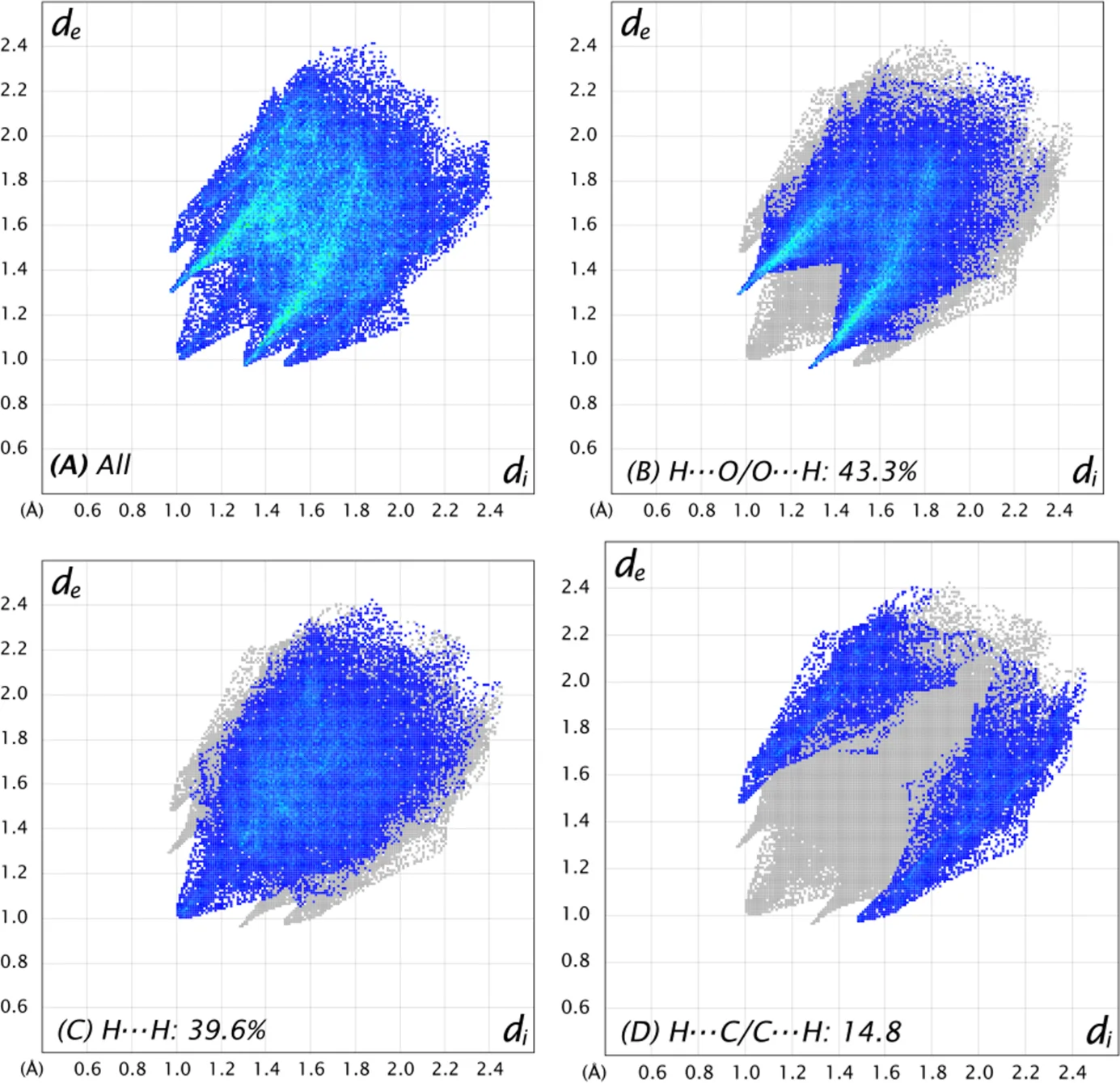
Two-dimensional Hirshfeld Plots of all surface contacts (A) and of the greatest atomic contact contributors of (B) H⋯O/O⋯H contacts, (C) H⋯H contacts and (D) H⋯C contacts. The brighter blue color shows greater abundance of surface inter­actions.

**Figure 6:**
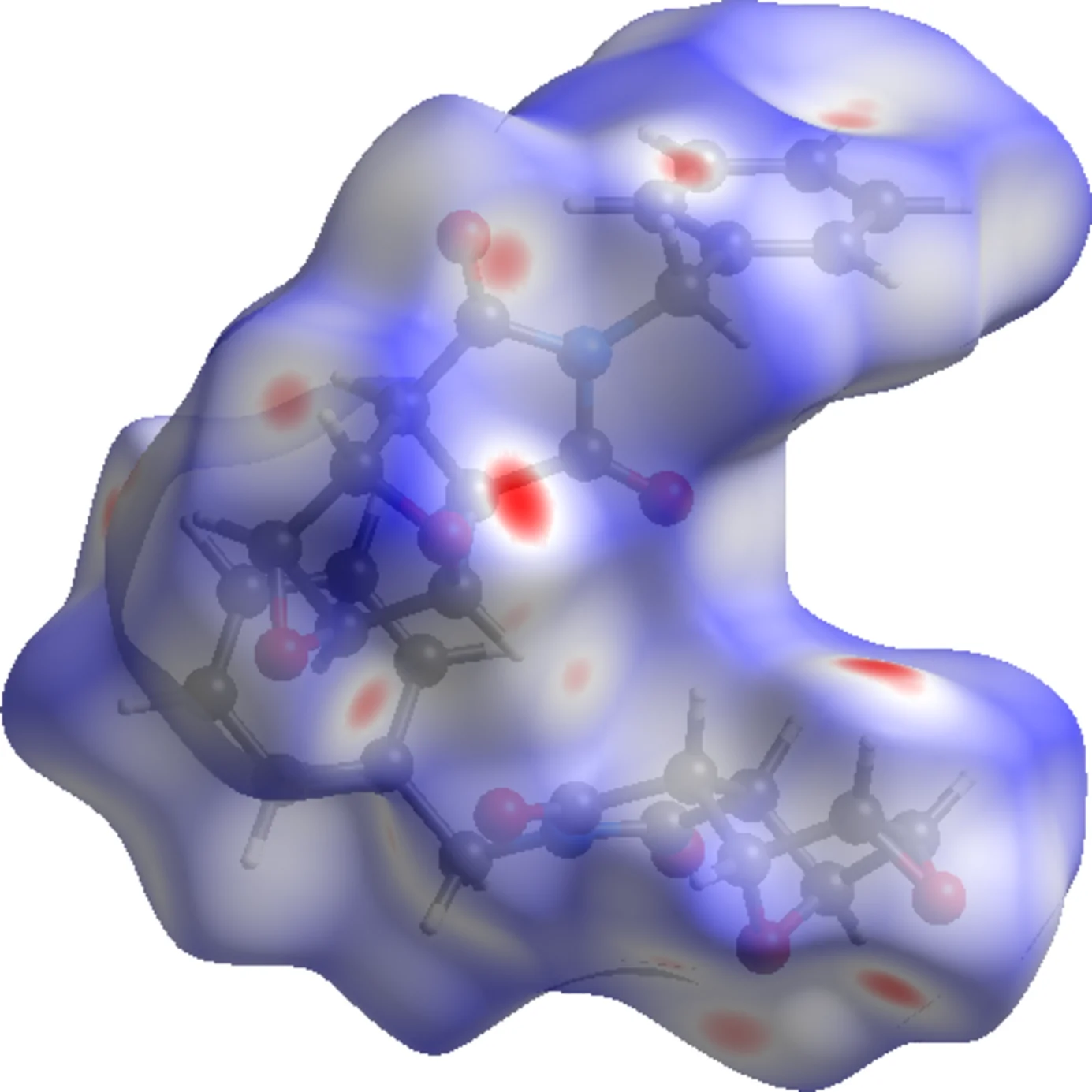
Three-dimensional Hirshfeld surface of the asymmetric unit of **4**, plotted over *d*_norm_ in the range −0.2533 to 1.3970 a.u. The darker red spheres show the greatest amount of inter­molecular surface inter­actions for specific atoms.

Further analyzing the surface contacts in the expanded crystal provides no evidence of classical hydrogen bonding that would explain the inter­molecular inter­actions. We conducted both a two- and a three-dimensional Hirshfeld analysis to probe the types of other inter­molecular inter­actions (such as Van der Waals) within the crystal of **4**, with details summarized in Table 1 ▸, Fig. 5 ▸ and Fig. 6 ▸. The majority of the surface atom contacts were assigned to H⋯O atoms (43.3%), H.·H atoms (39.6%) and H⋯C atoms (14.8%) as shown in Table 1 ▸ and two-dimensional Hirshfeld plots in Fig. 4 ▸. This is further shown by the three-dimensional Hirshfeld view, where the strongest indication of surface inter­actions (in red) is positioned around the epoxide and furan oxygen atoms, and the axial H atoms in the tetra­cyclic core.

## 4. Database survey

A database survey (CSD, July 2026; Groom *et al.*, 2016 ▸) with the tetra­cyclic core and the corresponding stereochemistry observed in **4** revealed only one other structure (Ren *et al.* 2026 ▸, CCDC: 2486893), with an *N*-3,5-di­chloro­phenyl substituent rather than an *N*-benzyl substituent in **4**.

## 5. Synthesis and crystallization

All reactions were conducted under air in ambient conditions unless otherwise stated. All solvents were reagent grade and used as received. ^1^H and ^13^C{^1^H} NMR were conducted on a JEOL ECA-II 400 MHz and JEOL ECA-II 600 MHz spectrometers. All ^1^H and ^13^C{^1^H} NMR spectra were referenced to the residual solvent signal of CDCl_3_ at 7.26 and 77.16 ppm, respectively. IR spectra were obtained from a JASCO FT/IR-6600 spectrometer using the neat solid compounds. Melting point temperatures were collected on an OptiMelt MPA100 melting point apparatus.

*7-Oxabi­cyclo­[2.2.1]hept-5-ene-2,3-di­carb­oxy­lic anhydride (**1**)*

To a 100 mL round-bottomed flask (RBF) equipped with a septum and stir bar and containing maleic anhydride (8.0 g, 81.6 mmol, 3 equiv.) was added 40 mL cyclo­pentyl methyl ether (CPME). The mixture was externally warmed (318 K) until a solution was formed. After the solution was allowed to cool to room temperature, furan (2.0 mL, 27.5 mmol, 1 equiv.) was added by syringe. The sealed reaction mixture was allowed to stir at room temperature for 3 d at which time, the solid was collected by vacuum filtration and rinsed with diethyl ether (40 mL). Obtained after filtration was a white crystalline solid in 3.5 g (76% yield) and used without further purification. ^1^H NMR (400 MHz, CDCl_3_); 6.58 (*s*, 2H, =CH), 5.47 (*s*, 2H, –CHO–), 3.18 (*s*, 2H, CH).

*3-(Benzyl­carbamo­yl)-5,6-di­hydro-7-oxabi­cyclo­[2.2.1]heptane-2-carb­oxy­lic acid (**2**)*

7-Oxabi­cyclo­[2.2.1]hept-5-ene-2,3-di­carb­oxy­lic anhydride (**1**) (1.5 g, 8.9 mmol, 1.0 equiv.) was added to a 100 mL RBF equipped with a magnetic stir bar and septum. Di­chloro­methane (40 mL) was next added to the RBF at room temperature and placed under a blanket of argon. Benzyl­amine (1.0 mL, 9.4 mmol, 1.05 equiv.) was dissolved in di­chloro­methane (5 mL) and as a solution, added to the RBF using a disposable Pasteur pipet. The resulting mixture which began forming a precipitant within 5 min of adding the amine was allowed to stir overnight while maintaining the blanket of argon. After adding diethyl ether (20 mL) to the reaction mixture and allowing the slurry to stir for approximately 10 min at room temperature, the solid was isolated by filtration and rinsing with diethyl ether (50 mL). Obtained after filtration was a white crystalline solid in 2.2 g (91% yield) and used without further purification. ^1^H NMR (400 MHz, CDCl_3_); 7.32–7.31 (*m*, 5H), 6.79 (*br s*, 1H), 6.48 (*d*, *J* = 5.2 Hz, 1H), 6.40 (*d*, *J* = 4.8 Hz, 1H), 5.33 (*s*, 1H), 5.08 (*s*, 1H), 4.43 (*dd*, *J* = 14.0, 6.0 Hz, 1H), 4.31 (*dd*, *J* = 14.0, 5.2 Hz, 1H), 2.90 (*d*, *J* = 9.2 Hz, 1H), 2.82 (*d*, *J* = 8.4 Hz, 1H).

*N-Benzyl-5,6-di­hydro-7-oxabi­cyclo­[2.2.1]heptane-2,3-dicarboximide (**3**)*

After the addition of 3-(benzyl­carbamo­yl)-5,6-di­hydro-7-oxabi­cyclo­[2.2.1]heptane-2-carb­oxy­lic acid (**2**) (139 mg, 0.51 mmol, 1.0 equiv.), di­chloro­methane (DCM) (4.5 mL), di­methyl­formamide (DMF) (0.50 mL), and 1-hy­droxy­benzotriazole (HOBt) (86 mg, 0.56 mmol, 1.1 equiv.) to a 10 mL RBF, the solution was externally cooled using an ice bath and placed under a blanket of Ar. A solution of di­cyclo­hexyl­carbodi­imide (DCC) (116 mg, 0.56 mmol, 1.1 equiv.) in 5 mL of DCM was added dropwise directly into the RBF by syringe. The reaction mixture was allowed to gradually warm to room temperature under a blanket of argon and stir overnight at which time the reaction mixture was filtered through a Celite pad as part of a disposable Pasteur pipet. After the pipet was rinsed with DCM (5 mL), the solution was concentrated *in vacuo* and purified by column chromatography (SiO_2_ (10 mm x 80 mm); gradient system EtOAc:hexa­nes 1:4 (50 mL), 1:2 (50 mL), 1:1 (50 mL); 10 mL fractions collecting tubes 5-14). The isolated material after removal of the volatiles in vacuo was 103 mg [0.40 mmol (79% yield using 3-(benzyl­carbamo­yl)-5,6-di­hydro-7-oxabi­cyclo­[2.2.1]heptane-2-carb­oxy­lic acid (**2**) as limiting reactant)]; white solid; ^1^H NMR (400 MHz, CDCl_3_); 7.32–7.31 (*m*, 5H), 6.52 (*s*, 2H), 5.30 (*s*, 2H), 4.65 (*s*, 2H), 2.87 (*s*, 2H).

*N-Benzyl-5,6-ep­oxy-7-oxabi­cyclo­[2.2.1]heptane-2,3-dicarb­oximide (* **4** *)*

After the addition of *N*-benzyl-5,6-di­hydro-7-oxabi­cyclo­[2.2.1]heptane-2,3-dicarboximide (**3**) (266 mg, 1.04 mmol, 1.0 equiv.) and di­chloro­methane (DCM) (2 mL) to a 30 mL (RBF), the solution was externally cooled using an ice bath and placed under a blanket of Ar. A suspension of *m*-chloro­perbenzoic acid (*m*CPBA) (359 mg, 2.08 mmol, 2.0 equiv.) in 3 mL of DCM was added after approximately five minutes dropwise directly into the RBF using a disposable Pasteur pipet. [Note: The actual amount weighed was 553 mg factoring the 65% purity.] The vial which contained the *m*CPBA was rinsed and transferred to the RBF using approximately 0.5 mL of DCM. The reaction mixture was allowed to gradually warm to room temperature under a blanket of Ar and stir overnight at which time the excess oxidant was quenched upon addition of a saturated aqueous solution of sodium sulfite (5 mL). After stirring at room temperature for 10 min, the organic phase was washed multiple times to remove 3-chloro­benzoic acid as the reaction was found to go to completion when working with 2.0 equiv. *m*CPBA using a saturated aqueous solution of sodium bicarbonate (3 × 5 mL). The organic layer was next washed with brine, dried over anhydrous magnesium sulfate, and concentrated in vacuo. To obtain analytically pure material, the material was chromatographed (SiO_2_ (40 mm × 90 mm); gradient system EtOAc:hexa­nes 1:1 (100 mL), 2:1 (100 mL), 4:1 (200 mL); 20 mL fractions collecting tubes 9–18). The isolated material after removal of the volatiles in vacuo was 275 mg [1.0 mmol (97% yield using *N*-benzyl-5,6-di­hydro-7-oxabi­cyclo­[2.2.1]heptane-2,3-dicarboximide (**3**) as limiting reactant)]; white solid; m.p. 462–463 K. IR (neat, ATR) cm^−1^: 2968 (*w*), 1773 (*w*), 1955 (*s*), 1395 (*m*), 1175 (*s*), 1016 (*s*), 859 (*s*), 741 (*s*), 695 (*s*), 585 (*s*).

^1^H NMR (CDCl_3_, 600 MHz): δ 7.30–7.26 (*m*, 5H), 4.84 (*s*, 2H), 4.64 (s, 2H), 3.50 (*s*, 2H), 2.99 (*s*, 2H). ^13^C{^1^H} NMR (CDCl_3_, 150 MHz): d 175.1, 135.1, 128.7, 128.1, 127.9, 76.7, 49.7, 47.9, 42.8. TLC: Silica gel 60 *F_254_* (2:1 ethyl acetate/hexa­nes); *R_f_* = 0.3.

*Crystallization and mounting of **4***

After dissolving 59 mg of **4** in 1.1 mL of ethyl acetate, 0.4 mL of the solution were placed into a 1.5 mL tube. The tube was then carefully placed into a 20 mL screw-capped vial charged with approximately 6 mL hexa­nes. After sealing the setup, large colorless block-like and plate crystals formed after 24 h. Crystals were taken directly from mother liquor using a spatula with paratone oil, and dispersed into more paratone oil. The crystal was put on a mounting loop, put on the goniometer head and frozen under a stream of LN_2_ (143 K) while data collection was conducted.

## 6. Refinement

Crystal data collection and structure refinement details are shown and summarized in Table 2 ▸. The crystal data was refined as a two-component inversion twin (twin law: [−1, 0, 0, 0, −1, 0, 0, 0, −1]). Due to poor agreement of observed values to calculated values, 5 reflections (1 1 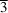, 0 2 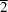, 1 2 2, 2 5 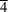, 4 4 1) were omitted in the refinement. H atoms were attached to their parent atoms geometrically and constrained to ride on their parent atoms.

## Supplementary Material

Crystal structure: contains datablock(s) I. DOI: 10.1107/S2056989026009205/yy2025sup1.cif

Structure factors: contains datablock(s) I. DOI: 10.1107/S2056989026009205/yy2025Isup2.hkl

CCDC reference: 2585299

Additional supporting information:  crystallographic information; 3D view; checkCIF report

## Figures and Tables

**Table 1:** Listed surface atom contacts and their percentage contribution to total contacts

Atom contact	Contribution (%)
H⋯O/O⋯H	43.3
H⋯H	39.6
H⋯C/C⋯H	14.8
H⋯N/N⋯H	1.1
O⋯O	0.7
C⋯O/O⋯C	0.6

**Table 2:** Experimental details

Crystal data	
Chemical formula	C_15_H_13_NO_4_
*M*_r_	271.26
Crystal system, space group	Orthorhombic, *Pca*2_1_
Temperature (K)	143
*a*, *b*, *c* (Å)	11.078 (5), 11.988 (5), 18.432 (8)
*V* (Å^3^)	2447.9 (19)
*Z*	8
Radiation type	Mo *K*α
μ (mm^−1^)	0.11
Crystal size (mm)	0.31 × 0.20 × 0.14

Data collection	
Diffractometer	Rigaku Pilatus 200K
Absorption correction	Numerical (Busing et al., 1957 ▸; Coppens *et al.*, 1965 ▸)
*T*_min_, *T*_max_	0.985, 0.991
No. of measured, independent and observed [*I* > 2σ(*I*)] reflections	37607, 5347, 4234
*R*_int_	0.044
(sin θ/λ)_max_ (Å^−1^)	0.649

Refinement	
*R*[*F*^2^ > 2σ(*F*^2^)], *wR*(*F*^2^), *S*	0.034, 0.077, 0.92
No. of reflections	5347
No. of parameters	362
No. of restraints	1
H-atom treatment	H-atom parameters constrained
Δρ_max_, Δρ_min_ (e Å^−3^)	0.25, −0.18
Absolute structure	Refined as an inversion twin

Computer programs: *CrystalClear-SM Expert* (Rigaku, 2016 ▸), *SHELXT* (Sheldrick, 2015*a* ▸), *SHELXL2019/2* (Sheldrick 2015*b* ▸), *ShelXle* (Hübschle *et al.*, 2011 ▸), *OLEX2* (Dolomanov *et al.*, 2009 ▸) and CrystalExplorer (Spackman *et al.*, 2021 ▸).

## supplementary crystallographic information

### N-Benzyl-5,6-epoxy-7-oxabicyclo[2.2.1]heptane-2,3-dicarboximide Crystal data

**Table:**

C_15_H_13_NO_4_	*D*_x_ = 1.472 Mg m^−^^3^
*M**_r_* = 271.26	Mo *K*α radiation, λ = 0.71075 Å
Orthorhombic, *P**c**a*2_1_	Cell parameters from 3248 reflections
*a* = 11.078 (5) Å	θ = 3.3–27.5°
*b* = 11.988 (5) Å	µ = 0.11 mm^−^^1^
*c* = 18.432 (8) Å	*T* = 143 K
*V* = 2447.9 (19) Å^3^	Block-like, colorless
*Z* = 8	0.31 × 0.20 × 0.14 mm
*F*(000) = 1136	

### N-Benzyl-5,6-epoxy-7-oxabicyclo[2.2.1]heptane-2,3-dicarboximide Data collection

**Table:**

Rigaku Pilatus 200K diffractometer	4234 reflections with *I* > 2σ(*I*)
Radiation source: Rotating Anode	*R*_int_ = 0.044
profile data from ω–scans	θ_max_ = 27.5°, θ_min_ = 3.3°
Absorption correction: numerical (Busing et al., 1957; Coppens *et al.*, 1965)	*h* = −14→14
*T*_min_ = 0.985, *T*_max_ = 0.991	*k* = −15→11
37607 measured reflections	*l* = −23→23
5347 independent reflections	

### N-Benzyl-5,6-epoxy-7-oxabicyclo[2.2.1]heptane-2,3-dicarboximide Refinement

**Table:**

Refinement on *F*^2^	Secondary atom site location: difference Fourier map
Least-squares matrix: full	Hydrogen site location: inferred from neighbouring sites
*R*[*F*^2^ > 2σ(*F*^2^)] = 0.034	H-atom parameters constrained
*wR*(*F*^2^) = 0.077	*w* = 1/[σ^2^(*F*_o_^2^) + (0.0499*P*)^2^] where *P* = (*F*_o_^2^ + 2*F*_c_^2^)/3
*S* = 0.92	(Δ/σ)_max_ < 0.001
5347 reflections	Δρ_max_ = 0.25 e Å^−^^3^
362 parameters	Δρ_min_ = −0.18 e Å^−^^3^
1 restraint	Absolute structure: Refined as an inversion twin
Primary atom site location: dual	

### N-Benzyl-5,6-epoxy-7-oxabicyclo[2.2.1]heptane-2,3-dicarboximide Special details

**Table:**

Geometry. All esds (except the esd in the dihedral angle between two l.s. planes) are estimated using the full covariance matrix. The cell esds are taken into account individually in the estimation of esds in distances, angles and torsion angles; correlations between esds in cell parameters are only used when they are defined by crystal symmetry. An approximate (isotropic) treatment of cell esds is used for estimating esds involving l.s. planes.
Refinement. Refined as a 2-component inversion twin.

### N-Benzyl-5,6-epoxy-7-oxabicyclo[2.2.1]heptane-2,3-dicarboximide Fractional atomic coordinates and isotropic or equivalent isotropic displacement parameters (Å^2^)

**Table:**

	*x*	*y*	*z*	*U*_iso_*/*U*_eq_	
O1	−0.11945 (18)	0.50167 (16)	0.64782 (12)	0.0307 (5)	
O2	−0.06787 (16)	0.49189 (14)	0.51523 (11)	0.0192 (4)	
O1A	0.13975 (17)	1.02313 (17)	0.34982 (11)	0.0303 (5)	
O3	0.22361 (16)	0.36364 (15)	0.43955 (10)	0.0289 (4)	
O2A	0.18982 (15)	1.02605 (14)	0.48285 (9)	0.0199 (4)	
O4	0.07162 (16)	0.70987 (14)	0.40423 (9)	0.0260 (4)	
O3A	0.48025 (15)	1.12960 (14)	0.56683 (10)	0.0262 (4)	
O4A	0.32226 (16)	0.78153 (15)	0.58545 (10)	0.0278 (4)	
N1	0.16171 (19)	0.53901 (17)	0.40756 (12)	0.0206 (5)	
N1A	0.40913 (17)	0.95429 (17)	0.59153 (11)	0.0194 (4)	
C1	0.0028 (3)	0.4713 (3)	0.63137 (15)	0.0309 (7)	
H1	0.060539	0.450985	0.670915	0.037*	
C2	−0.0374 (2)	0.5851 (2)	0.62233 (13)	0.0248 (6)	
H2	−0.008089	0.645671	0.655227	0.030*	
C1A	0.2652 (2)	1.0441 (2)	0.36676 (14)	0.0281 (6)	
H1A	0.325082	1.061602	0.327794	0.034*	
C3	0.0226 (2)	0.4313 (2)	0.55475 (14)	0.0232 (6)	
H3	0.021379	0.348487	0.548042	0.028*	
C2A	0.2146 (2)	0.9333 (2)	0.37433 (13)	0.0236 (6)	
H2A	0.238647	0.871535	0.340889	0.028*	
C4	−0.0389 (2)	0.6008 (2)	0.54095 (13)	0.0186 (5)	
H4	−0.092383	0.661999	0.522908	0.022*	
C3A	0.2857 (2)	1.0810 (2)	0.44490 (14)	0.0221 (5)	
H3A	0.291239	1.163377	0.452598	0.027*	
C5	0.1396 (3)	0.4906 (2)	0.52978 (15)	0.0212 (7)	
H5	0.205042	0.486004	0.567012	0.025*	
C4A	0.2109 (2)	0.9161 (2)	0.45570 (14)	0.0190 (5)	
H4A	0.152631	0.858144	0.472839	0.023*	
C6	0.0943 (2)	0.61035 (19)	0.51891 (13)	0.0187 (5)	
H6	0.139769	0.666128	0.548568	0.022*	
C5A	0.3964 (2)	1.0131 (2)	0.47013 (15)	0.0172 (6)	
H5A	0.464537	1.016201	0.434641	0.021*	
C7	0.1816 (2)	0.4530 (2)	0.45617 (15)	0.0209 (5)	
C6A	0.3429 (2)	0.89612 (19)	0.47726 (12)	0.0173 (5)	
H6A	0.383732	0.841027	0.444741	0.021*	
C8	0.1057 (2)	0.6304 (2)	0.43823 (13)	0.0195 (5)	
C7A	0.4354 (2)	1.0435 (2)	0.54662 (14)	0.0203 (5)	
C9	0.1925 (2)	0.5348 (2)	0.33081 (13)	0.0278 (6)	
H9A	0.118912	0.548682	0.301724	0.033*	
H9B	0.222156	0.459083	0.318786	0.033*	
C8A	0.3552 (2)	0.8660 (2)	0.55583 (13)	0.0190 (5)	
C10	0.2880 (2)	0.6195 (2)	0.31034 (13)	0.0209 (6)	
C9A	0.4347 (2)	0.9512 (2)	0.66915 (13)	0.0251 (6)	
H9AA	0.360486	0.929416	0.695550	0.030*	
H9AB	0.457937	1.026788	0.685621	0.030*	
C11	0.2762 (2)	0.6842 (2)	0.24819 (13)	0.0230 (6)	
H11	0.208689	0.674147	0.217123	0.028*	
C10A	0.5341 (2)	0.8706 (2)	0.68755 (13)	0.0217 (6)	
C12	0.3623 (2)	0.7627 (2)	0.23165 (12)	0.0240 (6)	
H12	0.353218	0.807033	0.189268	0.029*	
C11A	0.6367 (2)	0.8630 (2)	0.64473 (15)	0.0325 (7)	
H11A	0.643953	0.908179	0.602619	0.039*	
C13	0.4613 (2)	0.7782 (2)	0.27534 (14)	0.0250 (6)	
H13	0.519662	0.833509	0.263768	0.030*	
C12A	0.7284 (3)	0.7901 (2)	0.66289 (16)	0.0373 (7)	
H12A	0.798131	0.785277	0.633028	0.045*	
C14	0.4751 (2)	0.7121 (2)	0.33665 (13)	0.0257 (6)	
H14	0.544037	0.721240	0.366729	0.031*	
C13A	0.7196 (2)	0.7244 (2)	0.72402 (15)	0.0296 (7)	
H13A	0.782799	0.674282	0.736315	0.036*	
C15	0.3898 (2)	0.6336 (2)	0.35402 (13)	0.0248 (6)	
H15	0.399988	0.588673	0.396010	0.030*	
C14A	0.6184 (2)	0.7320 (2)	0.76712 (12)	0.0255 (6)	
H14A	0.612238	0.687524	0.809637	0.031*	
C15A	0.5263 (2)	0.8034 (2)	0.74899 (13)	0.0219 (6)	
H15A	0.456375	0.807033	0.778732	0.026*	

### N-Benzyl-5,6-epoxy-7-oxabicyclo[2.2.1]heptane-2,3-dicarboximide Atomic displacement parameters (Å^2^)

**Table:**

	*U*^11^	*U*^22^	*U*^33^	*U*^12^	*U*^13^	*U*^23^
O1	0.0230 (10)	0.0431 (13)	0.0261 (11)	−0.0020 (8)	0.0078 (9)	0.0068 (8)
O2	0.0153 (9)	0.0201 (10)	0.0223 (10)	−0.0016 (7)	−0.0032 (8)	−0.0013 (7)
O1A	0.0248 (11)	0.0368 (11)	0.0294 (12)	0.0011 (9)	−0.0088 (10)	0.0067 (9)
O3	0.0239 (10)	0.0221 (10)	0.0408 (11)	0.0029 (8)	−0.0028 (8)	−0.0060 (8)
O2A	0.0144 (9)	0.0215 (10)	0.0238 (10)	0.0039 (7)	0.0005 (8)	−0.0034 (7)
O4	0.0361 (10)	0.0176 (10)	0.0243 (9)	−0.0017 (8)	−0.0021 (8)	0.0039 (7)
O3A	0.0225 (9)	0.0225 (10)	0.0335 (10)	−0.0046 (8)	0.0003 (7)	−0.0047 (8)
O4A	0.0331 (10)	0.0214 (10)	0.0291 (10)	−0.0024 (8)	−0.0034 (8)	0.0064 (8)
N1	0.0227 (11)	0.0190 (12)	0.0201 (11)	−0.0023 (10)	0.0026 (9)	−0.0018 (9)
N1A	0.0196 (11)	0.0203 (12)	0.0183 (11)	0.0020 (10)	−0.0011 (9)	−0.0005 (9)
C1	0.0184 (13)	0.051 (2)	0.0235 (16)	0.0057 (13)	0.0007 (12)	0.0142 (13)
C2	0.0180 (13)	0.0338 (16)	0.0227 (13)	−0.0095 (12)	0.0010 (10)	−0.0020 (11)
C1A	0.0213 (14)	0.0369 (17)	0.0261 (16)	−0.0007 (13)	−0.0019 (11)	0.0088 (12)
C3	0.0209 (13)	0.0184 (13)	0.0304 (15)	0.0000 (11)	0.0035 (11)	0.0079 (11)
C2A	0.0222 (14)	0.0314 (16)	0.0173 (13)	0.0045 (11)	−0.0014 (10)	−0.0011 (11)
C4	0.0184 (12)	0.0165 (13)	0.0208 (12)	−0.0012 (10)	−0.0024 (10)	−0.0048 (9)
C3A	0.0210 (13)	0.0179 (13)	0.0275 (14)	−0.0051 (11)	−0.0004 (11)	0.0060 (11)
C5	0.0175 (15)	0.0249 (15)	0.0211 (16)	0.0007 (10)	−0.0036 (12)	0.0030 (10)
C4A	0.0183 (12)	0.0176 (13)	0.0211 (12)	−0.0018 (10)	−0.0018 (10)	−0.0012 (10)
C6	0.0177 (12)	0.0182 (14)	0.0202 (12)	−0.0039 (10)	−0.0026 (10)	0.0002 (10)
C5A	0.0129 (13)	0.0209 (14)	0.0179 (15)	0.0008 (10)	0.0007 (12)	0.0019 (10)
C7	0.0113 (11)	0.0197 (15)	0.0318 (14)	−0.0027 (11)	−0.0014 (11)	0.0016 (11)
C6A	0.0159 (12)	0.0169 (13)	0.0192 (12)	0.0016 (10)	0.0004 (10)	−0.0041 (10)
C8	0.0187 (13)	0.0182 (13)	0.0217 (12)	−0.0066 (11)	−0.0018 (10)	−0.0016 (10)
C7A	0.0118 (11)	0.0228 (15)	0.0264 (13)	0.0026 (11)	0.0003 (10)	−0.0047 (11)
C9	0.0336 (15)	0.0322 (17)	0.0176 (13)	−0.0053 (13)	0.0027 (11)	−0.0065 (12)
C8A	0.0163 (12)	0.0179 (14)	0.0230 (13)	0.0051 (10)	−0.0005 (10)	−0.0002 (10)
C10	0.0215 (13)	0.0220 (14)	0.0192 (13)	0.0003 (10)	0.0041 (10)	−0.0056 (10)
C9A	0.0297 (15)	0.0281 (16)	0.0176 (13)	0.0058 (13)	−0.0043 (11)	−0.0019 (11)
C11	0.0217 (13)	0.0290 (15)	0.0182 (13)	0.0043 (12)	0.0000 (11)	−0.0049 (11)
C10A	0.0220 (14)	0.0248 (14)	0.0182 (13)	−0.0013 (11)	−0.0037 (10)	−0.0030 (10)
C12	0.0267 (14)	0.0280 (16)	0.0173 (15)	0.0046 (12)	0.0053 (12)	0.0015 (11)
C11A	0.0259 (14)	0.0421 (18)	0.0295 (14)	0.0033 (13)	0.0058 (12)	0.0143 (13)
C13	0.0207 (14)	0.0252 (15)	0.0290 (15)	0.0010 (12)	0.0054 (11)	0.0001 (11)
C12A	0.0253 (15)	0.0483 (19)	0.0383 (17)	0.0042 (14)	0.0106 (12)	0.0181 (15)
C14	0.0208 (12)	0.0330 (15)	0.0232 (13)	0.0009 (12)	−0.0006 (10)	−0.0003 (11)
C13A	0.0201 (14)	0.0348 (17)	0.0340 (16)	0.0031 (12)	−0.0027 (12)	0.0079 (13)
C15	0.0270 (13)	0.0281 (15)	0.0195 (12)	0.0031 (12)	−0.0006 (10)	0.0037 (11)
C14A	0.0291 (15)	0.0255 (16)	0.0220 (15)	−0.0054 (12)	−0.0001 (12)	0.0046 (11)
C15A	0.0188 (12)	0.0295 (15)	0.0173 (13)	−0.0035 (12)	0.0008 (10)	−0.0039 (11)

### N-Benzyl-5,6-epoxy-7-oxabicyclo[2.2.1]heptane-2,3-dicarboximide Geometric parameters (Å, º)

**Table:**

O1—C2	1.431 (3)	C4A—C6A	1.534 (3)
O1—C1	1.435 (3)	C4A—H4A	1.0000
O2—C4	1.426 (3)	C6—C8	1.512 (3)
O2—C3	1.436 (3)	C6—H6	1.0000
O1A—C2A	1.432 (3)	C5A—C7A	1.519 (4)
O1A—C1A	1.446 (3)	C5A—C6A	1.528 (4)
O3—C7	1.207 (3)	C5A—H5A	1.0000
O2A—C4A	1.429 (3)	C6A—C8A	1.499 (3)
O2A—C3A	1.432 (3)	C6A—H6A	1.0000
O4—C8	1.201 (3)	C9—C10	1.514 (3)
O3A—C7A	1.204 (3)	C9—H9A	0.9900
O4A—C8A	1.207 (3)	C9—H9B	0.9900
N1—C8	1.380 (3)	C10—C11	1.389 (3)
N1—C7	1.384 (3)	C10—C15	1.396 (3)
N1—C9	1.456 (3)	C9A—C10A	1.505 (3)
N1A—C8A	1.382 (3)	C9A—H9AA	0.9900
N1A—C7A	1.383 (3)	C9A—H9AB	0.9900
N1A—C9A	1.459 (3)	C11—C12	1.374 (4)
C1—C2	1.445 (4)	C11—H11	0.9500
C1—C3	1.508 (4)	C10A—C11A	1.387 (4)
C1—H1	1.0000	C10A—C15A	1.392 (3)
C2—C4	1.512 (3)	C12—C13	1.373 (4)
C2—H2	1.0000	C12—H12	0.9500
C1A—C2A	1.448 (4)	C11A—C12A	1.381 (4)
C1A—C3A	1.524 (4)	C11A—H11A	0.9500
C1A—H1A	1.0000	C13—C14	1.389 (4)
C3—C5	1.548 (4)	C13—H13	0.9500
C3—H3	1.0000	C12A—C13A	1.378 (4)
C2A—C4A	1.514 (4)	C12A—H12A	0.9500
C2A—H2A	1.0000	C14—C15	1.371 (4)
C4—C6	1.534 (3)	C14—H14	0.9500
C4—H4	1.0000	C13A—C14A	1.377 (4)
C3A—C5A	1.543 (3)	C13A—H13A	0.9500
C3A—H3A	1.0000	C15—H15	0.9500
C5—C7	1.504 (4)	C14A—C15A	1.373 (4)
C5—C6	1.534 (3)	C14A—H14A	0.9500
C5—H5	1.0000	C15A—H15A	0.9500
			
C2—O1—C1	60.56 (19)	C7A—C5A—C3A	112.3 (2)
C4—O2—C3	97.91 (19)	C6A—C5A—C3A	101.7 (2)
C2A—O1A—C1A	60.39 (17)	C7A—C5A—H5A	112.5
C4A—O2A—C3A	97.55 (18)	C6A—C5A—H5A	112.5
C8—N1—C7	113.4 (2)	C3A—C5A—H5A	112.5
C8—N1—C9	122.1 (2)	O3—C7—N1	123.9 (3)
C7—N1—C9	124.5 (2)	O3—C7—C5	127.9 (2)
C8A—N1A—C7A	113.5 (2)	N1—C7—C5	108.2 (2)
C8A—N1A—C9A	122.1 (2)	C8A—C6A—C5A	105.6 (2)
C7A—N1A—C9A	124.5 (2)	C8A—C6A—C4A	112.0 (2)
O1—C1—C2	59.59 (17)	C5A—C6A—C4A	101.75 (19)
O1—C1—C3	114.6 (2)	C8A—C6A—H6A	112.3
C2—C1—C3	103.7 (2)	C5A—C6A—H6A	112.3
O1—C1—H1	120.8	C4A—C6A—H6A	112.3
C2—C1—H1	120.8	O4—C8—N1	123.8 (2)
C3—C1—H1	120.8	O4—C8—C6	127.8 (2)
O1—C2—C1	59.84 (17)	N1—C8—C6	108.3 (2)
O1—C2—C4	114.0 (2)	O3A—C7A—N1A	124.3 (2)
C1—C2—C4	103.6 (2)	O3A—C7A—C5A	127.6 (2)
O1—C2—H2	121.0	N1A—C7A—C5A	108.1 (2)
C1—C2—H2	121.0	N1—C9—C10	112.5 (2)
C4—C2—H2	121.0	N1—C9—H9A	109.1
O1A—C1A—C2A	59.31 (17)	C10—C9—H9A	109.1
O1A—C1A—C3A	113.4 (2)	N1—C9—H9B	109.1
C2A—C1A—C3A	103.5 (2)	C10—C9—H9B	109.1
O1A—C1A—H1A	121.3	H9A—C9—H9B	107.8
C2A—C1A—H1A	121.3	O4A—C8A—N1A	123.9 (2)
C3A—C1A—H1A	121.3	O4A—C8A—C6A	127.7 (2)
O2—C3—C1	102.3 (2)	N1A—C8A—C6A	108.4 (2)
O2—C3—C5	101.59 (19)	C11—C10—C15	118.9 (2)
C1—C3—C5	104.7 (2)	C11—C10—C9	121.0 (2)
O2—C3—H3	115.5	C15—C10—C9	120.1 (2)
C1—C3—H3	115.5	N1A—C9A—C10A	112.3 (2)
C5—C3—H3	115.5	N1A—C9A—H9AA	109.1
O1A—C2A—C1A	60.30 (17)	C10A—C9A—H9AA	109.1
O1A—C2A—C4A	113.5 (2)	N1A—C9A—H9AB	109.1
C1A—C2A—C4A	103.3 (2)	C10A—C9A—H9AB	109.1
O1A—C2A—H2A	121.1	H9AA—C9A—H9AB	107.9
C1A—C2A—H2A	121.1	C12—C11—C10	120.0 (2)
C4A—C2A—H2A	121.1	C12—C11—H11	120.0
O2—C4—C2	102.6 (2)	C10—C11—H11	120.0
O2—C4—C6	101.35 (18)	C11A—C10A—C15A	118.4 (2)
C2—C4—C6	105.2 (2)	C11A—C10A—C9A	120.9 (2)
O2—C4—H4	115.3	C15A—C10A—C9A	120.6 (2)
C2—C4—H4	115.3	C13—C12—C11	121.1 (2)
C6—C4—H4	115.3	C13—C12—H12	119.4
O2A—C3A—C1A	102.6 (2)	C11—C12—H12	119.4
O2A—C3A—C5A	101.52 (19)	C12A—C11A—C10A	120.4 (2)
C1A—C3A—C5A	104.5 (2)	C12A—C11A—H11A	119.8
O2A—C3A—H3A	115.5	C10A—C11A—H11A	119.8
C1A—C3A—H3A	115.5	C12—C13—C14	119.2 (2)
C5A—C3A—H3A	115.5	C12—C13—H13	120.4
C7—C5—C6	105.3 (2)	C14—C13—H13	120.4
C7—C5—C3	113.0 (2)	C13A—C12A—C11A	120.5 (3)
C6—C5—C3	101.2 (2)	C13A—C12A—H12A	119.8
C7—C5—H5	112.2	C11A—C12A—H12A	119.8
C6—C5—H5	112.2	C15—C14—C13	120.4 (2)
C3—C5—H5	112.2	C15—C14—H14	119.8
O2A—C4A—C2A	103.0 (2)	C13—C14—H14	119.8
O2A—C4A—C6A	102.10 (18)	C14A—C13A—C12A	119.5 (2)
C2A—C4A—C6A	104.6 (2)	C14A—C13A—H13A	120.3
O2A—C4A—H4A	115.1	C12A—C13A—H13A	120.3
C2A—C4A—H4A	115.1	C14—C15—C10	120.4 (2)
C6A—C4A—H4A	115.1	C14—C15—H15	119.8
C8—C6—C4	110.64 (19)	C10—C15—H15	119.8
C8—C6—C5	104.5 (2)	C15A—C14A—C13A	120.4 (2)
C4—C6—C5	102.1 (2)	C15A—C14A—H14A	119.8
C8—C6—H6	112.9	C13A—C14A—H14A	119.8
C4—C6—H6	112.9	C14A—C15A—C10A	120.8 (2)
C5—C6—H6	112.9	C14A—C15A—H15A	119.6
C7A—C5A—C6A	104.5 (2)	C10A—C15A—H15A	119.6
			
C2—O1—C1—C3	−92.0 (3)	C3—C5—C7—N1	108.6 (2)
C1—O1—C2—C4	92.3 (2)	C7A—C5A—C6A—C8A	−0.5 (3)
C3—C1—C2—O1	110.7 (2)	C3A—C5A—C6A—C8A	−117.5 (2)
O1—C1—C2—C4	−110.0 (2)	C7A—C5A—C6A—C4A	116.5 (2)
C3—C1—C2—C4	0.6 (3)	C3A—C5A—C6A—C4A	−0.5 (2)
C2A—O1A—C1A—C3A	−92.2 (2)	O2A—C4A—C6A—C8A	78.5 (2)
C4—O2—C3—C1	51.7 (2)	C2A—C4A—C6A—C8A	−174.3 (2)
C4—O2—C3—C5	−56.3 (2)	O2A—C4A—C6A—C5A	−33.8 (2)
O1—C1—C3—O2	30.2 (3)	C2A—C4A—C6A—C5A	73.3 (2)
C2—C1—C3—O2	−32.3 (3)	C7—N1—C8—O4	−173.7 (2)
O1—C1—C3—C5	135.8 (2)	C9—N1—C8—O4	4.5 (4)
C2—C1—C3—C5	73.3 (2)	C7—N1—C8—C6	5.3 (3)
C1A—O1A—C2A—C4A	92.4 (2)	C9—N1—C8—C6	−176.4 (2)
C3A—C1A—C2A—O1A	109.5 (2)	C4—C6—C8—O4	64.3 (3)
O1A—C1A—C2A—C4A	−109.7 (2)	C5—C6—C8—O4	173.5 (2)
C3A—C1A—C2A—C4A	−0.2 (3)	C4—C6—C8—N1	−114.7 (2)
C3—O2—C4—C2	−51.4 (2)	C5—C6—C8—N1	−5.5 (3)
C3—O2—C4—C6	57.2 (2)	C8A—N1A—C7A—O3A	178.5 (2)
O1—C2—C4—O2	−31.2 (3)	C9A—N1A—C7A—O3A	−1.5 (4)
C1—C2—C4—O2	31.6 (2)	C8A—N1A—C7A—C5A	−0.8 (3)
O1—C2—C4—C6	−136.8 (2)	C9A—N1A—C7A—C5A	179.3 (2)
C1—C2—C4—C6	−74.1 (2)	C6A—C5A—C7A—O3A	−178.4 (2)
C4A—O2A—C3A—C1A	51.5 (2)	C3A—C5A—C7A—O3A	−69.0 (3)
C4A—O2A—C3A—C5A	−56.4 (2)	C6A—C5A—C7A—N1A	0.8 (3)
O1A—C1A—C3A—O2A	30.2 (3)	C3A—C5A—C7A—N1A	110.2 (2)
C2A—C1A—C3A—O2A	−31.8 (3)	C8—N1—C9—C10	66.0 (3)
O1A—C1A—C3A—C5A	135.8 (2)	C7—N1—C9—C10	−116.0 (3)
C2A—C1A—C3A—C5A	73.8 (3)	C7A—N1A—C8A—O4A	−178.0 (2)
O2—C3—C5—C7	−79.3 (2)	C9A—N1A—C8A—O4A	1.9 (3)
C1—C3—C5—C7	174.6 (2)	C7A—N1A—C8A—C6A	0.4 (3)
O2—C3—C5—C6	32.9 (2)	C9A—N1A—C8A—C6A	−179.6 (2)
C1—C3—C5—C6	−73.3 (2)	C5A—C6A—C8A—O4A	178.5 (2)
C3A—O2A—C4A—C2A	−51.9 (2)	C4A—C6A—C8A—O4A	68.5 (3)
C3A—O2A—C4A—C6A	56.4 (2)	C5A—C6A—C8A—N1A	0.1 (2)
O1A—C2A—C4A—O2A	−30.8 (3)	C4A—C6A—C8A—N1A	−109.8 (2)
C1A—C2A—C4A—O2A	32.4 (3)	N1—C9—C10—C11	−135.3 (2)
O1A—C2A—C4A—C6A	−137.2 (2)	N1—C9—C10—C15	44.8 (3)
C1A—C2A—C4A—C6A	−74.0 (2)	C8A—N1A—C9A—C10A	69.9 (3)
O2—C4—C6—C8	75.3 (2)	C7A—N1A—C9A—C10A	−110.1 (3)
C2—C4—C6—C8	−178.1 (2)	C15—C10—C11—C12	−1.8 (4)
O2—C4—C6—C5	−35.4 (2)	C9—C10—C11—C12	178.2 (2)
C2—C4—C6—C5	71.2 (2)	N1A—C9A—C10A—C11A	41.6 (3)
C7—C5—C6—C8	3.8 (3)	N1A—C9A—C10A—C15A	−139.7 (2)
C3—C5—C6—C8	−114.0 (2)	C10—C11—C12—C13	0.5 (4)
C7—C5—C6—C4	119.1 (2)	C15A—C10A—C11A—C12A	0.1 (4)
C3—C5—C6—C4	1.3 (2)	C9A—C10A—C11A—C12A	178.8 (3)
O2A—C3A—C5A—C7A	−76.8 (2)	C11—C12—C13—C14	0.9 (4)
C1A—C3A—C5A—C7A	176.8 (2)	C10A—C11A—C12A—C13A	−0.3 (5)
O2A—C3A—C5A—C6A	34.4 (2)	C12—C13—C14—C15	−1.2 (4)
C1A—C3A—C5A—C6A	−72.0 (2)	C11A—C12A—C13A—C14A	−0.1 (4)
C8—N1—C7—O3	175.9 (2)	C13—C14—C15—C10	−0.1 (4)
C9—N1—C7—O3	−2.3 (4)	C11—C10—C15—C14	1.6 (4)
C8—N1—C7—C5	−2.7 (3)	C9—C10—C15—C14	−178.5 (2)
C9—N1—C7—C5	179.0 (2)	C12A—C13A—C14A—C15A	0.8 (4)
C6—C5—C7—O3	−179.6 (2)	C13A—C14A—C15A—C10A	−1.0 (4)
C3—C5—C7—O3	−70.0 (3)	C11A—C10A—C15A—C14A	0.5 (4)
C6—C5—C7—N1	−0.9 (3)	C9A—C10A—C15A—C14A	−178.2 (2)

## References

[bb1] Alves, T. V. & Fernández, I. (2023). *Org. Biomol. Chem.* **21**, 7767–7775. 10.1039/d3ob01343j 37698053

[bb2] Busing, W. R. & Levy, H. A. (1957). *Acta Cryst.* **10**, 180–182.

[bb3] Chattopadhyay, D., Swingle, M. R., Salter, E. A., Wood, E., D’Arcy, B., Zivanov, C. N., Abney, K., Musiyenko, A., Rusin, S. F., Kettenbach, A., Yet, L., Schroeder, C. E., Golden, J. E., Dunham, W. H., Gingras, A. C., Banerjee, S., Forbes, D. C., Wierzbicki, A. & Honkanen, R. E. (2016). *Biochem. Pharmacol.* **109**, 14–26. 10.1016/j.bcp.2016.03.011 PMC4853234 27002182

[bb4] Coppens, P., Leiserowitz, L. & Rabinovich, D. (1965). *Acta Cryst.* **18**, 1035–1038.

[bb5] Dolomanov, O. V., Bourhis, L. J., Gildea, R. J., Howard, J. A. K. & Puschmann, H. (2009). *J. Appl. Cryst.* **42**, 339–341.

[bb6] Groom, C. R., Bruno, I. J., Lightfoot, M. P. & Ward, S. C. (2016). *Acta Cryst.* B**72**, 171–179. 10.1107/S2052520616003954 PMC4822653 27048719

[bb7] Hill, H. P., Baker, N. C., Nguyen, I. B. & Forbes, D. C. (2022). *Journal of Undergraduate Chemistry Research* **21**, 19–24.

[bb8] Hübschle, C. B., Sheldrick, G. M. & Dittrich, B. (2011). *J. Appl. Cryst.* **44**, 1281–1284. 10.1107/S0021889811043202 PMC3246833 22477785

[bb9] Lawley, H. K., Baxter, B. N., Lopansri, C. N., Marmande, M. H., Mayeaux, K. N., Orr, L. A. & Forbes, D. C. (2026). *Molbank* M2124.

[bb10] Ren, Z., Yang, P., Chen, H., Feng, Y., Wu, E., Wang, Z., Chen, Y., Gao, Q. & Wang, J. (2026). *Pestic. Biochem. Physiol.* **219**, 107040. 10.1016/j.pestbp.2026.107040 41831908

[bb11] Rigaku (2016). *CrystalClear-SM Expert*. Rigaku Corporation, Tokyo, Japan.

[bb12] Scott, K. A., McCluskey, A., Njardarson, J. T. & Wang, W. (2026). *Med. Chem.* **17**, 743–767. 10.1039/d5md00917k PMC12772682 41502824

[bb13] Sheldrick, G. M. (2015*a*). *Acta Cryst.* A**71**, 3–8.

[bb14] Sheldrick, G. M. (2015*b*). *Acta Cryst.* C**71**, 3–8.

[bb15] Spackman, P. R., Turner, M. J., McKinnon, J. J., Wolff, S. K., Grimwood, D. J., Jayatilaka, D. & Spackman, M. A. (2021). *J. Appl. Cryst.* **54**, 1006–1011. 10.1107/S1600576721002910 PMC8202033 34188619

